# An assessment of morphological and pathological changes in paravertebral muscle degeneration using imaging and histological analysis: a cross-sectional study

**DOI:** 10.1186/s12891-021-04734-3

**Published:** 2021-10-08

**Authors:** Ding-Chao Zhu, Jia-Hao Lin, Jia-Jing Xu, Qiang Guo, Yi-Han Wang, Chao Jiang, Hui-Gen Lu, Yao-Sen Wu

**Affiliations:** 1 Department of Orthopedics, Department of Orthopaedic Surgery, The Second Affiliated Hospital and Yuying Children’s Hospital of Wenzhou Medical University, 109# Xueyuan Road, Wenzhou, 325000 China; 2 Zhejiang Provincial Key Laboratory of Orthopaedics, Wenzhou, China; 3 The Second School of Medicine, Wenzhou Medical University, Wenzhou, China; 4 Department of Orthopaedic Surgery, The Second Hospital of Jiaxing, Jiaxing, China

**Keywords:** Low back pain, Paravertebral muscle, Magnetic resonance imaging, Fatty degeneration, Inflammatory

## Abstract

**Background:**

The high signal of paravertebral muscle (PVM) on T2-weighted image (T2WI) is usually considered to be fatty degeneration. However, it is difficult to distinguish inflammatory edema from fatty degeneration on T2WI. The purpose of this study was to identify different types of PVM high signal in patients with low back pain (LBP) through magnetic resonance imaging (MRI) and histology.

**Methods:**

Seventy patients with LBP underwent MRI. The signal change of multifidus both on T2WI and fat suppression image (FSI) was quantified by Image J. Furthermore, 25 of the 70 patients underwent surgery for degenerative lumbar disease and their multifidus were obtained during the operation. Histological analysis of the samples was performed by HE staining.

**Result:**

Three types of PVM signal changes were identified from the MRI. Type 1 (*n* = 36) indicated fatty degeneration characterized by a high signal on T2WI and low signal on FSI. High signal on both T2WI and FSI, signifying type 2 meant inflammatory edema (*n* = 9). Type 3 (*n* = 25) showed high signal on T2WI and partial signal suppression on FSI, which meant a combination of fatty degeneration and inflammatory edema. Histological results were consistent with MRI. Among the 25 patients who underwent surgery, type 1 (*n* = 14) showed adipocytes infiltration, type 2 (*n* = 3) showed inflammatory cells infiltration and type 3 (*n* = 8) showed adipocytes and inflammatory cells infiltration.

**Conclusion:**

From our results, there are three types of pathological changes in patients with PVM degeneration, which may help to decide on targeted treatments for LBP.

## Background

Low back pain (LBP) is a complex and multifactorial disorder commonly found in middle-aged and elderly individuals [1]. Previous studies have demonstrated that a number of lesions, such as nerve root compression, disc degeneration, modic change, facet joint osteoarthritis, and spinal stenosis are all associated with LBP [2–4]. However, the main source and related mechanism of LBP are currently unknown. Paravertebral muscle (PVM) degeneration has attracted more attention from researchers. A number of studies have shown the relationship between LBP and PVM degeneration. In the process of PVM degeneration, normal muscle fiber morphology changes and is replaced by adipose tissue resulting in a decrease in the stability of the vertebral column [5]. This may be one of the important reasons behind LBP. Additionally, Parkkola et al. [6] found increased fatty infiltration (FI) and reduced cross-sectional area (CSA) of PVM in patients with LBP compared with the control group. Storheim et al. [7] found that patients with less fatty infiltration of PVM had better results after the treatment of LBP. However, the pathological process and specific mechanism of PVM from normal to degeneration are still unclear.

At present, the measurement of PVM morphology has been an effective method for reflecting FI and has been used for investigations into the etiology of LBP [8]. Magnetic resonance imaging (MRI) as a reliable measurement method, can clearly identify the characteristics of different groups of muscle and the difference in signal intensity between fat and muscle. Previous studies were mainly carried out on the T2-weighted image (T2WI). However, fat and liquid have a relatively longer T2-relaxation time and higher signal intensity on T2WI than other soft tissue such as normal skeletal muscle. Therefore, there is a possibility that fatty degeneration of PVM may be combined with inflammatory edema. In this study, we suspected that there might be different types of PVM pathological changes and that the high signal of PVM on T2WI might not only be fatty degeneration, but also inflammation edema. Studying PVM degeneration on both T2WI and FSI could easily distinguish the signal intensity between fat and edema.

A biopsy can help us understand the microstructure of PVM degeneration in patients with LBP. Bahar Shahidi et al. took multifidus muscle biopsy on 22 patients undergoing surgical treatment for the degenerative lumbar disease. They found high levels of muscle degeneration and inflammation and decreased vascularity [9]. Subsequently, Bahar Shahidi et al. found increased fibrogenic gene expression in the multifidus muscle of patients with chronic compared to acute LBP [10]. However, these studies employed a simple histological observation and genetic test but did not combine the histological changes in multifidus with imaging. Furthermore, although there have been previous studies about the imaging and histological changes of damaged PVM after lumbar surgery [11], studies combining imaging and histological changes of non-surgical degenerative PVM are still rare. Therefore, in the present study, we mainly studied PVM degeneration in the natural process at a microscopic and macroscopic level using MRI and histological analyses.

This study found three types of pathology in the process of PVM degeneration, and each corresponded to different imaging. Our study provides a basis for the personalized treatment of PVM-induced LBP.

## Methods

### Patients

A cross-sectional study was performed. A total of 70 patients with LBP who consecutively came to the Second Affiliated Hospital of Wenzhou Medical University from December 2018 to December 2019 were included in the study. Only patients with LBP, no prior spine surgery, no systemic inflammatory disease, no acute trauma, neoplasm, or infection were included in this study. The following data were recorded for each LBP patient: sex, age, body mass index (BMI) and symptoms duration, etc. This study were approved by the Second Affiliated Hospital of Wenzhou Medical University Ethics Committee and followed the guidelines of the Helsinki Declaration. All participants signed written informed consent before the experiments.

### Imaging evaluation

All patients underwent MRI examinations. The MRI system was a 1.5 Tesla Imaging System (GE Health care Milwaukee, WI, USA). Cross-sectional views of lumbar were obtained using a fast spin-echo sequence system for T2WI and FSI. The slice width was 4 mm and the inter-slice gap was 1 mm. The acquisition matrix was 512 × 256. The sequence parameters were a repetition time of 2300 ms and an echo time of 99 ms for T2WI.

**Fig. 1:**
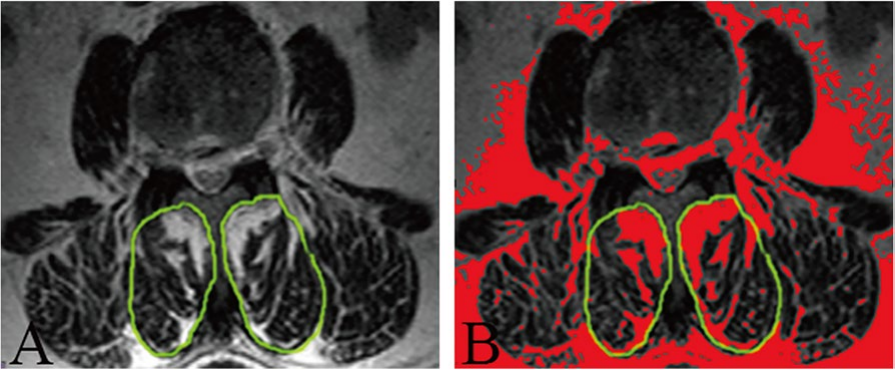
Demonstration of threshold technology of Image J. A female patient aged 66 years old: High signal of multifidus muscle surrounded by green line colored in white on T2WI (**A**); High signal of multifidus muscle surrounded by green line colored in red using the threshold technique of Image J on T2WI (**B**)

Images were analyzed with Image J software (U.S. National Institutes of Health, Bethesda, MD) and stored on the computer. Two spine surgeon doctors with more than 10 years of work experience completed the assessment. Cross-sectional views of T2WI and FSI were taken at the corresponding spinal degeneration segment level (L4/S1 mainly). The slice of the spinal degeneration segment was considered as the research object. The lumbar PVM of interest in this study was multifidus. The signal intensity of the multifidus was examined using the following steps on T2WI and FSI. The scale pixel was first set, and each image converted to a grayscale 8-bit image. Image J software was then used to outline CSA of the multifidus. The signal intensity was then quantified using a threshold technique. The high signal area in the 8-bit image was colored in red using the threshold tool of the program (Fig. 1) [12, 13].

### Pathological examination

A total of 25 patients underwent surgery for degenerative lumbar spine pathology. During their operation, multifidus about 1 cm by 1 cm were cut from diseased segment. Multifidus was fixed, dehydrated, impregnated, embedded in paraffin to form wax block and sectioned for haematoxylin and eosin (HE) staining. Histological analysis was made by one experienced pathologist, blinded to the MRI finding and objective of this study.

### Statistical analysis

Statistical analysis was performed using SPSS version 24.0 (SPSS Inc., Chicago, USA). All continuous data were described as mean ± standard deviation. An analysis of variance was carried out to detect differences in age and BMI among the different types of PVM degeneration. An analysis of variance and the Kruskal-Wallis test were performed to detect differences in duration and distribution among the different types. The sex in different types was analyzed using the Chi-square test. *P* values less than 0.05 were considered statistically significant.

## Result

### MRI evaluation of multifidus

**Fig. 2:**
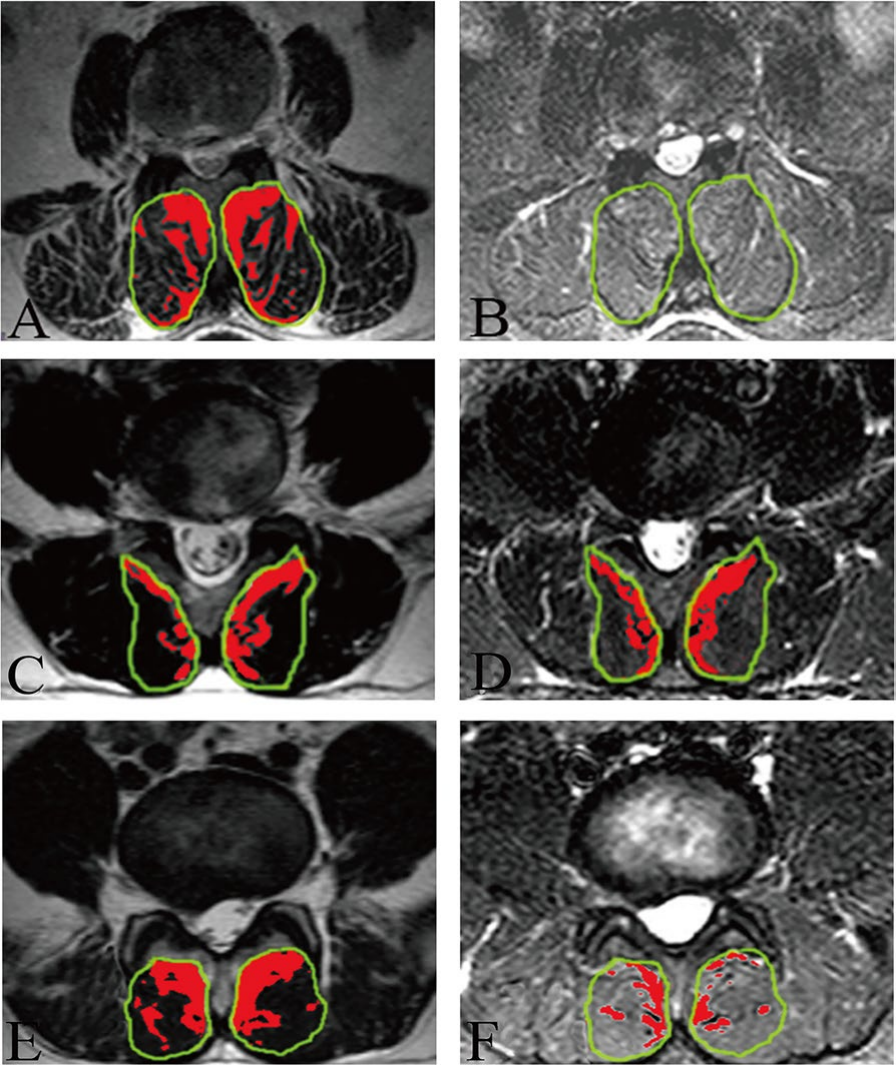
Representative T2WI and FSI in patients with different PVM degeneration. A female patient aged 66 years old (**A** and **B**): High signal (red area) of multifidus (green line) was displayed using the threshold technique of Image J on T2WI and FSI. A male patient aged 25 years old (**C** and **D**): High signal (red area) of multifidus (green line) was shown using the threshold technique of Image J on T2WI and FSI. A male patient aged 43 years old (**E** and **F**): High signal (red area) of multifidus (green line) was shown using the threshold technique of Image J on T2WI and FSI

The demographic data of the 70 patients are presented in Table 1. A total of 36 patients (51.42%, 57.33 ± 7.24 years) showed high signal on T2WI and low signal on FSI, which indicated fatty degeneration (Type 1, Fig. 2A, B). Nine patients (12.86%, 26.00 ± 5.12 years) showed high signal on both T2WI and FSI, which signified inflammatory edema (Type 2, Fig. 2C, D). High signal on T2WI and partial signal suppression on FSI, indicating a combination of fatty degeneration and inflammatory edema (Type 3, Fig. 2E, F) was observed in 25 patients (35.71%, 43.08 ± 5.79 years). Therefore, MRI examination showed three different types of PVM high signal: fatty degeneration, inflammatory edema, and a combination of fatty degeneration and inflammatory edema.

**Table 1:** Demographic data of patients in the MRI evaluation

	Type 1	Type 2	Type 3	*p value*
Cases	36 (51.42%)	9 (12.86%)	25 (35.71%)	
Sex (M:F)	15:21	6:3	11:14	0.424
Age (years)	57.33 ± 7.24	26.00 ± 5.12	43.08 ± 5.79	< 0.001*
BMI (kg/m^2^)	28.71 ± 3.40	23.46 ± 2.72	26.60 ± 2.78	< 0.001*

BMI: body mass index

*indicates significant difference

### Histological evaluation of multifidus

**Fig. 3:**
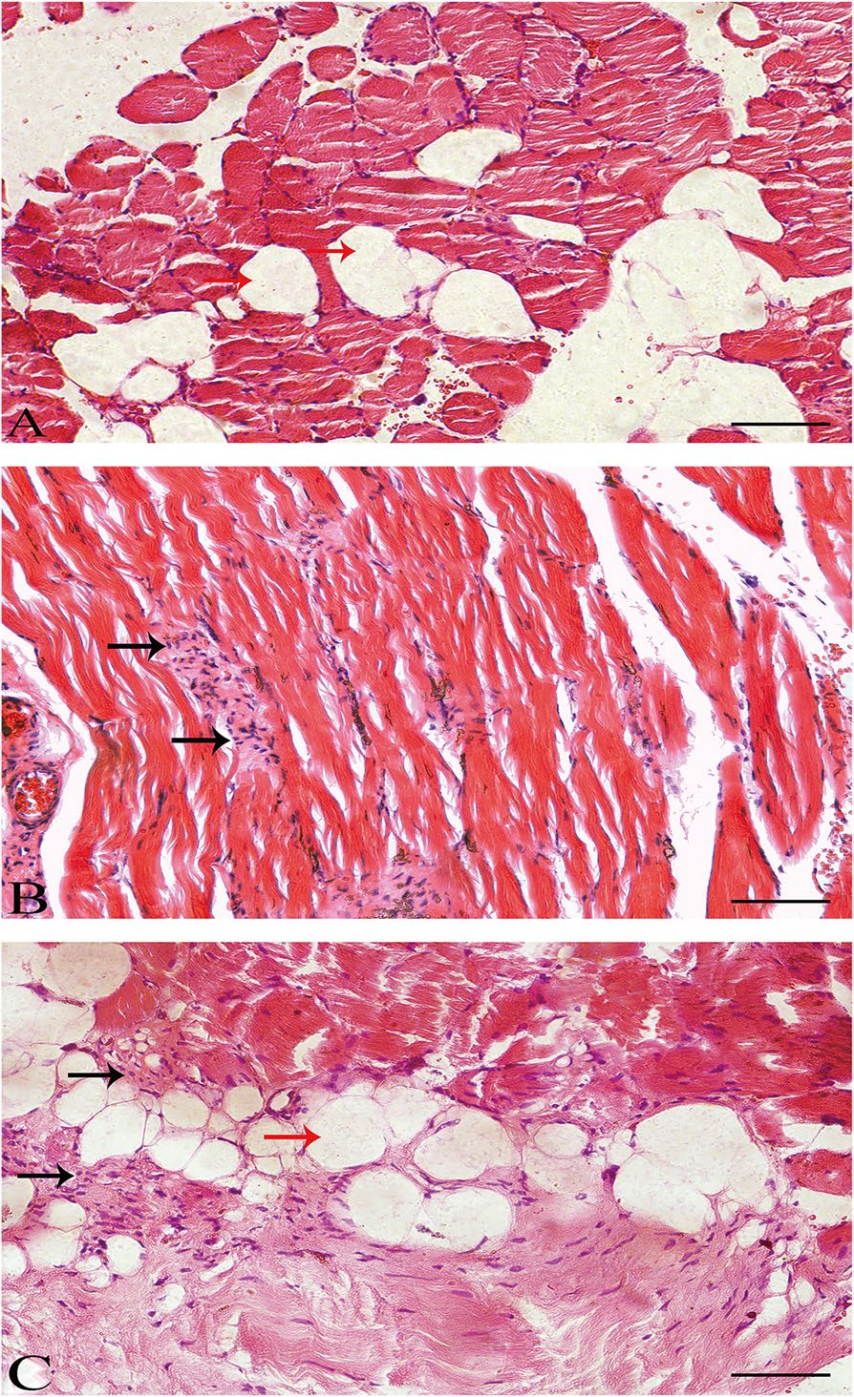
Representative histological sections in patients with PVM degeneration. A female patient aged 66 years old (**A**): Adipocytes infiltration of multifidus muscle, the red arrow indicates adipocytes. A male patient aged 25 years old (**B**): Inflammatory cells infiltration of multifidus muscle without adipocytes, black arrow indicates inflammatory cells (mainly neutrophils). A male patient aged 43 years old (**C**): Adipocytes and inflammatory cells infiltration, red and black arrows indicate adipocytes and inflammatory cells (mainly lymphocytes), respectively. (scale bar =100 μm)

The demographic information of the 25 surgical patients is listed in Table 2. From the histological analyses, adipocytes infiltration was observed in 14 patients (56.00%, 60.14 ± 6.27 years). Adipocytes could be seen in muscle tissue by HE staining and the muscle cell nuclei were squeezed into periphery without inflammatory cells infiltration (Fig. 3A). A total of 3 patients (12.00%, 27.00 ± 4.36 years) showed inflammatory cells infiltration. HE staining showed inflammatory cells (mainly neutrophils) in muscle tissue without adipocytes (Fig. 3B). Finally, 8 patients (32.00%, 45.88 ± 6.15 years) showed both adipocytes and inflammatory cells infiltration. The HE staining showed both adipocytes and inflammatory cells (mainly lymphocytes) in muscle tissue, light staining of inflammation edema area, and tissue gap narrowed or disappeared (Fig. 3C). Therefore, histological examination showed three different types of PVM degeneration: adipocytes infiltration, inflammatory cells infiltration and a combination of adipocytes and inflammatory cells infiltration.

**Table 2:** Demographic data of patients in the histological evaluation

	Adipocytes infiltration	Inflammatory cells infiltration	Adipocytes and inflammatory cells infiltration	*p value*
Cases	14 (56.00%)	3 (12.00%)	8 (32.00%)	
Sex (M:F)	6:8	2:1	5:3	
Age (years)	60.14 ± 6.27	27.00 ± 4.36	45.88 ± 6.15	< 0.001*
BMI (kg/m^2^)	30.82 ± 3.12	25.18 ± 2.12	29.44 ± 3.03	0.025*

BMI: body mass index

*indicates significant difference

**Fig. 4:**
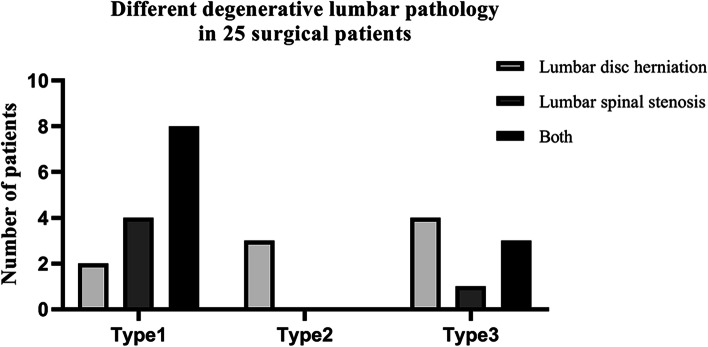
Degenerative lumbar pathology of 25 surgical patients. There were 2 patients with lumbar disc herniation, 4 patients with lumbar spinal stenosis, and 8 patients with lumbar disc herniation combined with lumbar spinal stenosis in type 1 (56.00%); All three patients in type 2 (12.00%) presented lumbar disc herniation; Type 3 patients (32.00%) included 4 of lumbar disc herniation, 1 of lumbar spinal stenosis, and 3 of lumbar disc herniation combined with lumbar spinal stenosis

Histological results of these 25 patients were consistent with their imaging. The 14 patients with adipocytes infiltration showed type 1 on imaging; the 3 patients with inflammatory cells infiltration showed type 2 and the 8 patients with both adipocytes and inflammatory cells infiltration showed type 3 on imaging. The proportion of different degenerative lumbar disease such as lumbar disc herniation (9, 36.00%), spinal stenosis (5, 20.00%), and a combination of the above both (11, 44.00%) in 25 surgical patients with different types were shown in Fig. 4.

### Demographic data evaluation

For patients of the different types of MRI evaluation, age (*p* < 0.001) and BMI (p < 0.001) indicated significant difference, while sex (*p* = 0.424) indicated no statistical difference (Table 1). For patients of the different types of histological evaluation, age (p < 0.001) and BMI (*p* = 0.025) indicated significant difference (Table 2). In addition, there were a statistical difference in the duration and distribution (p < 0.001, Table 3). The duration of type 1 patients mainly were longer than 12 weeks (58.33%), all type 2 patients lasted for less than 4 weeks (100%) and type 3 patients were mostly between 4 to 12 weeks (52.00%).

**Table 3:** Disease duration of patients with different types

	Type 1	Type 2	Type 3	*p value*
Duration (weeks)	23.15 ± 19.62	1.68 ± 1.26	8.55 ± 6.12	< 0.001*
Range	1.43, 92.57	0.14, 3.86	0.57, 26.71	
				< 0.001*
Less than 4 weeks	3 (8.33%)	9 (100%)	8 (32.00%)	
4 to 12 weeks	12 (33.33%)	0	13 (52.00%)	
More than 12 weeks	21 (58.33%)	0	4 (16.00%)	

The duration of less than 4 weeks manifests as acute LBP, 4 to 12 weeks manifests as subacute LBP and more than 12 weeks manifests as chronic LBP

*indicates significant difference

## Discussion

The PVM mainly comprises psoas, erector spinae, and multifidus muscle and is considered to have two functions: to stabilize and move the lumbar vertebral column. Kalichman et al. found that fatty degeneration in the PVM was common in adults and was strongly associated with LBP [5]. Therefore, studying PVM can help to discover the potential mechanism of lumbar instability and LBP. Moreover, Guo et al. found no significant difference in psoas or erector spinae between the patients with LBP and normal people [14]. Barker et al. reported multifidus as the largest and most medial PVM that is significantly stronger in normal persons than LBP patients and is sensitive to pathological changes [15]. This indicates that multifidus might be more involved in maintaining spinal stability and preventing chronic LBP.

MRI, computerized tomography (CT) and ultrasound are ideal methods for assessing the morphology of PVM. Many researchers have inclined to use MRI to analyze PVM in patients with lumbar diseases due to its clear muscular contour and high-fat resolution [16]. Hu et al. advised MRI rather than CT for the measurement of CSA and FI by comparing the intra- and inter-reliability [8]. Fortin et al. found that an increase in fat was caused by age and BMI in a 15-years longitudinal MRI study [17]. Previous studies mainly evaluated the degree of fat degeneration through signal intensity on T2WI. But since both fat and liquid show high signal on T2WI, measurement on T2WI cannot clearly distinguish between fatty degeneration and inflammatory edema of PVM. Thus, combining T2WI and FSI could provide a reasonable contrast between fat and liquid.

Previous, several MRI quantitative sequences like diffusion tensor imaging (DTI) or mDIXON-Quant sequence have been widely utilized in other literatures to quantify hepatic steatosis and is increasingly utilized to quantify intramuscular fat and bone marrow fat [18, 19]. DTI enables not only the characterization of back muscle fiber architecture, but also of muscle microstructure differences. Also, discrete and subclinical changes of the musculature caused by strenuous exercise can be identified with DTI that can remain undetected by using conventional T2WI or FSI sequences [20]. The mDIXON-Quant sequence has significant advantages such as rapid and volumetric data acquisition with visualization of anatomical structures and quantification of fat content in a region of interest [21]. However, a coregistration of these quantitative sequences images and fatty infiltration maps would be technically very demanding and was not performed in the context of this study.

CSA and FI are major indicators to evaluate PVM degeneration. Chon et al. found that CSA of multifidus was smaller in patients with chronic lumbar radiculopathy than in normal people [22]. Resorlu et al. observed that patients with ankylosing spondylitis had decreased CSA of multifidus, and it was related to the disease duration [23]. In a cross-sectional observation study, Barker et al. revealed that CSA of multifidus was smaller in patients with chronic LBP than in normal [15]. Moreover, Fortin et al. systematically reviewed the studies on the morphology of PVM in patients with LBP and the control group. They concluded that CSA of PVM in patients with LBP was significantly smaller than that in the control group [24]. Nonetheless, studies on the pathological mechanism of PVM degeneration are still rare. Therefore, in the present study, we mainly examined the pathological process for assessing PVM change.

The results of our study showed different types of signal changes in PVM. Type 1 included the patients with high signal on T2WI and low signal on FSI on MRI imaging and with adipocytes infiltration on histological analyses. Type 2 comprised of the patients with high signal on both T2WI and FSI and showed inflammatory cells (mainly neutrophils) infiltration. Type 3 included the patients with high signal on T2WI and partial signal suppression on FSI showed both adipocytes and inflammatory cells (mainly lymphocytes) infiltration. We further analyzed the age, BMI and disease duration and proportion of each type of patient. Among the 70 patients studied, type 1 had the largest proportion and mainly composed of middle-aged and elderly, the largest BMI, and chronic LBP. Type 2 had the least proportion and was mainly made up of the young, with the lowest BMI, and mainly acute LBP. The age, BMI, duration and proportion of type 3 cut across both type 1 and type 2. Inflammatory cells in type 2 were mainly neutrophils, which indicated acute inflammation whereas in type 3 the inflammation cells were mainly lymphocytes indicating chronic inflammation. Bahar Shahidi et al. found that muscle cells actively degenerated as opposed to simple atrophy, a process that may be facilitated by inflammation [9]. Hatice et al. found that chronic inflammation and cytokine-mediated fibrosis in patients with ankylosing spondylitis contributed to fatty degeneration and atrophy in the PVM [23]. In addition, Paul et al. reported a parallel increase in the expression of pro-inflammatory cytokines during the degeneration of PVM [25]. Therefore, we reasonably speculated that PVM degeneration in patients with LBP is a gradual process, and inflammation is an early pathological manifestation that might gradually transform into fatty degeneration.

Currently, many treatment modalities are used for LBP such as bed-rest, functional training of back muscle, physiotherapy, and medication, but their effectiveness are still inconclusive. Previous treatment strategies did not take into account the different pathological types of PVM degeneration. According to the imaging and histological results of this study, we speculate that in the absence of obvious spinal stenosis, instability and other pathological conditions, individualized conservative treatments are more likely to be effective for patients with different pathological changes of PVM. For patients with type 1 PVM degeneration, functional training for back muscle should be encouraged, because fatty degeneration is difficult to reverse with medication treatments or physiotherapy only. Bed-rest seems to not be of much benefit and may actually be detrimental because of fat deposit and muscle atrophy in these patients [26]. Storheim et al. reported that comprehensive training had a significant tendency of reversing the degeneration of PVM in patients with subacute LBP [27]. Hides et al. found that stability training could improve the CSA of multifidus in young athletes with LBP and reduce the pain [28]. However, according to the results of this study, we do not recommend high-intensity back muscle training for type 2 patients due to its acute inflammation. Bed-rest and non-steroidal anti-inflammatory drugs could be the best option, which can inhibit the progress of inflammation. Although most acute LBP is considered self-limiting, previous evidence found that a high percentage of individuals will experience recurrent symptoms leading to poor functional outcomes over time [29]. Bed-rest and drug treatment can effectively prevent type 2 patients from turning acute to chronic LBP. Because type 3 patients have a combined fatty degeneration and inflammation, we recommend that they should be treated in stages: firstly bed-rest and use of non-steroidal anti-inflammatory drugs to inhibit the progress of inflammation, then performing functional training. For many patients with acute LBP, clinicians may recommend two days of bed-rest rather than longer periods [30]. The results of this study can provide certain scientific guidance for the specific time of bed-rest. In individuals with persistent recurrence or chronic symptoms, muscle tissue is relatively slow to respond to traditional rehabilitation measures [31]. Therefore, understanding different types of pathological changes in PVM is crucial to determining appropriate treatment strategies.

There were some limitations in this study that need further discussion and investigations. The sample size in the study was small hence a need for further clinical research to support our personalized treatment methods for LBP and demographic data evaluation. Secondly, the study did not conduct a correlation analysis of clinical symptoms and pain scores, hence should be further studied. Since a large number of asymptomatic elderly individuals will have fatty infiltration and sarcopenia, it is necessary to use age or BMI matched asymptomatic controls for imaging comparison in the future. Based on this study, the specific transformation mechanism that may exist between fatty degeneration and inflammation in PVM needs further clarification.

## Conclusion

Based on the findings of this study, there are three types of pathology in the process of PVM degeneration, which may provide a basis for the personalized treatments of PVM-induced LBP. Besides, inflammation presents as an early pathological manifestation of PVM degeneration that gradually transforms into fatty degeneration.

## Data Availability

All data generated or analysed during this study are included in this published article.

## Abbreviations

PVM: Paravertebral muscle
T2WI: T2-weighted image
LBP: Low back pain
MRI: Magnetic resonance imaging
FSI: Fat suppression image
FI: Fatty infiltration
CSA: Cross-sectional area
BMI: Body mass index
HE: Haematoxylin and eosin
CT: Computerized tomography
DTI: Diffusion tensor imaging
